# Lifestyle Complexity and Dementia Risk: Examining Moderation by APOE Genotype and Mild Cognitive Impairment

**DOI:** 10.1093/geroni/igaa057.809

**Published:** 2020-12-16

**Authors:** Kyle Moored, Beth Snitz, Jeanine Parisi, Jeff Williamson, Annette Fitzpatrick, Steven DeKosky, Michelle Carlson

**Affiliations:** 1 Johns Hopkins School of Public Health, Baltimore, Maryland, United States; 2 University of Pittsburgh School of Medicine, Pittsburgh, Pennsylvania, United States; 3 Wake Forest School of Medicine, Winston-Salem, North Carolina, United States; 4 University of Washington, Seattle, Washington, United States; 5 University of Florida College of Medicine, Gainesville, Florida, United States; 6 Johns Hopkins Bloomberg School of Public Health, Baltimore, Maryland, United States

## Abstract

Prior studies suggest that the neuroprotective effect of physical exercise is moderated by APOE genotype and MCI status, but it remains unclear whether this extends to lifestyle complexity defined by a broader variety of physical, intellectual, and social activities. Participants were from the Ginkgo Evaluation of Memory (GEM) Study. We used 18 physical, intellectual, or social activities from the Lifestyle Activity Questionnaire. We performed latent class analysis to characterize subgroups with distinct activity response patterns and examined whether they have differential risk of incident dementia over time. A three-class model was chosen based on fit statistics and interpretability. Cox proportional hazards models, adjusted for potential demographic and health confounders, revealed that Class 1 (Highly intellectually/socially active) had a reduced risk of dementia compared to Class 3 (Less socially/less intellectually active; HR=.71, 95% CI: [.56,.88], p=.002). Class 2 (Socially/less intellectually active) did not differ in risk from Class 3 (HR=.90, 95% CI: [.73,1.1], p=.288). There was no evidence for effect modification for APOE e4 allele carriers (p’s>.05), but the protective association for Class 1 only held for those without prevalent MCI at baseline (HR=.74, 95% CI: [.56,.98], p=.033). Results showed that subgroups characterized by a greater variety of social and intellectual activities had reduced risk for dementia, but only for those without MCI. This implies that late-life lifestyle complexity may be most neuroprotective for those in the preclinical stages of decline. Results also suggested that lifestyle complexity may act through a cognitive reserve pathway unrelated to amyloid pathology.

